# Heparanase-Induced Activation of AKT Stabilizes β-Catenin and Modulates Wnt/β-Catenin Signaling during Herpes Simplex Virus 1 Infection

**DOI:** 10.1128/mBio.02792-21

**Published:** 2021-11-09

**Authors:** Lulia Koujah, Krishnaraju Madavaraju, Alex M. Agelidis, Chandrashekhar D. Patil, Deepak Shukla

**Affiliations:** a Department of Ophthalmology and Visual Sciences, University of Illinois at Chicago, Chicago, Illinois, USA; b Department of Microbiology and Immunology, University of Illinois at Chicago, Chicago, Illinois, USA; University of Calgary

**Keywords:** Wnt signaling, herpes simplex virus, host-pathogen interactions

## Abstract

Under pathological conditions like herpes simplex virus 1 (HSV-1) infection, host-pathogen interactions lead to major reconstruction of the host protein network, which contributes to the dysregulation of signaling pathways and disease onset. Of note is the upregulation of a multifunctional host protein, heparanase (HPSE), following infection, which serves as a mediator in HSV-1 replication. In this study, we identify a novel function of HPSE and highlight it as a key regulator of β-catenin signal transduction. The regulatory role of HPSE on the activation, nuclear translocation, and signal transduction of β-catenin disrupts cellular homeostasis and establishes a pathogenic environment that promotes viral replication. Under normal physiological conditions, β-catenin is bound to a group of proteins, referred to as the destruction complex, and targeted for ubiquitination and, ultimately, degradation. We show that virus-induced upregulation of HPSE leads to the activation of Akt and subsequent stabilization and activation of β-catenin through (i) the release of β-catenin from the destruction complex, and (ii) direct phosphorylation of β-catenin at Ser552. This study also provides an in-depth characterization of the proviral role of β-catenin signaling during HSV-1 replication using physiologically relevant cell lines and *in vivo* models of ocular infection. Furthermore, pharmacological inhibitors of this pathway generated a robust antiviral state against multiple laboratory and clinical strains of HSV-1. Collectively, our findings assign a novel regulatory role to HPSE as a driver of β-catenin signaling in HSV-1 infection.

## INTRODUCTION

Herpes simplex virus 1 (HSV-1) commonly infects roughly 3 billion people worldwide and is the leading cause of infection-related blindness in the developed world. HSV-1 infection induces a chronic immune-inflammatory response resulting in corneal scarring, thinning, and neovascularization. This leads to development of various ocular diseases such as herpes stromal keratitis. HSV-1 can also invade the central nervous system and lead to encephalitis, a relatively common cause of sporadic fetal encephalitis worldwide (1). Current antiviral treatments are able to block active viral replication or subside symptoms of infection; however, they lack the ability to completely abolish the virus from the host, primarily due to escape mutants or establishment of latent infection. Patients with herpetic ulcers are treated with nucleoside analogs and are always at risk of reactivation, which requires constant drug administration and leads to toxicity and evolution of drug-resistant viral strains. The ability to identify, study, and target host proteins that exhibit proviral roles will allow for greater therapeutic potential and scientific breadth.

Host-pathogen interactions lead to major reconstruction of the host protein network, which contributes to the dysregulation of signaling pathways and development of pathological environment. An example of this is virus-induced upregulation of heparanase (HPSE), a key mediator in HSV-1 replication. HPSE is the only known mammalian enzyme that can degrade heparan sulfate, an essential polysaccharide in the extracellular matrix. Our previous work highlights HPSE as a key driver of HSV pathogenesis primarily through the degradation of HS and promotion of virion release and viral spread (2–5). HPSE also participates in extracellular matrix (ECM) remodeling and has been implicated in the development and progression of a variety of pathologies such as tumor growth, angiogenesis, inflammation, and viral infections (6–8). Studies show that HPSE exhibits nonenzymatic activity by inducing signaling cascades to enhance phosphorylation of proteins such as Akt, ERK, p38, and Src (9, 10). These pathways exhibit cross talk with the Wnt/β-catenin signaling pathway through Akt-mediated phosphorylation of GSK-3β, a negative regulator of β-catenin, or direct phosphorylation and activation of β-catenin (11, 12). The Wnt/β-catenin pathway is extensively studied and recognized as having a major role in pathogenesis (13). However, a possible relationship between the two pathways, which may be particularly important in disease prognosis, remains poorly understood.

More recently, we demonstrated the multifunctional properties of HPSE and established important connections between its function and cellular proliferation and defense in response to viral infection (5). Curiously, our proteomic analysis of wild-type (WT) and Hpse-knockout (KO) mouse embryonic fibroblasts (MEFs) identified β-catenin as a differentially regulated hub protein that influences multiple host processes, including immune system processes, chromatin organization, and cell surface receptor signaling. Our unbiased proteomic studies thus raised the possibility of an important connection between HPSE and β-catenin signaling.

Under normal physiological conditions, β-catenin is either bound to the intracellular domain of E-cadherin to maintain structural integrity of the cell or is recognized by the destruction complex and targeted for degradation (14). Wnt protein secretion and binding to its cell surface receptor lead to the activation of the canonical Wnt signaling pathway, which results in the bypassing of β-catenin degradation, its accumulation in the cytoplasm, and subsequent nuclear translocation. In the nucleus, β-catenin binds to TCF/LEF and recruits transcriptional coactivators like CBP in order to regulate genes involved in cell survival and developmental processes (15).

We had recently reported that β-catenin is a driver of proviral signaling and that inhibition of β-catenin resulted in robust antiviral activity against HSV-1 infection (5). However, it was unexpected and puzzling that in the cells lacking HPSE, this pathway remained largely unperturbed. Therefore, in this study, we use HSV-1 infection to investigate the consequences of β-catenin signal transduction and the pathological contributions made by HPSE on β-catenin regulation. We provide new evidence that β-catenin signaling occurs following HSV-1 infection in a murine model of ocular infection to enhance cellular survival and viral replication. β-Catenin activity is promoted by virus-induced upregulation of HPSE, which serves to initiate a signaling cascade leading to the dissociation and stabilization of β-catenin from the destruction complex.

Finally, we delve into the therapeutic potential of targeting the β-catenin pathway. We show the robust antiviral properties of PRI-724, iCRT14, and KYA1797k in multiple cell lines and against various laboratory and clinical strains of HSV-1. With drugs targeting the β-catenin signal transduction pathway for therapeutic intervention already undergoing clinical trials for diseases like cancer and hepatitis B virus (HBV), there is great potential for its use as an antiviral against herpes infection (16–19).

## RESULTS

### HSV-1 infection causes β-catenin nuclear translocation and signal transduction.

**FIG 1:**
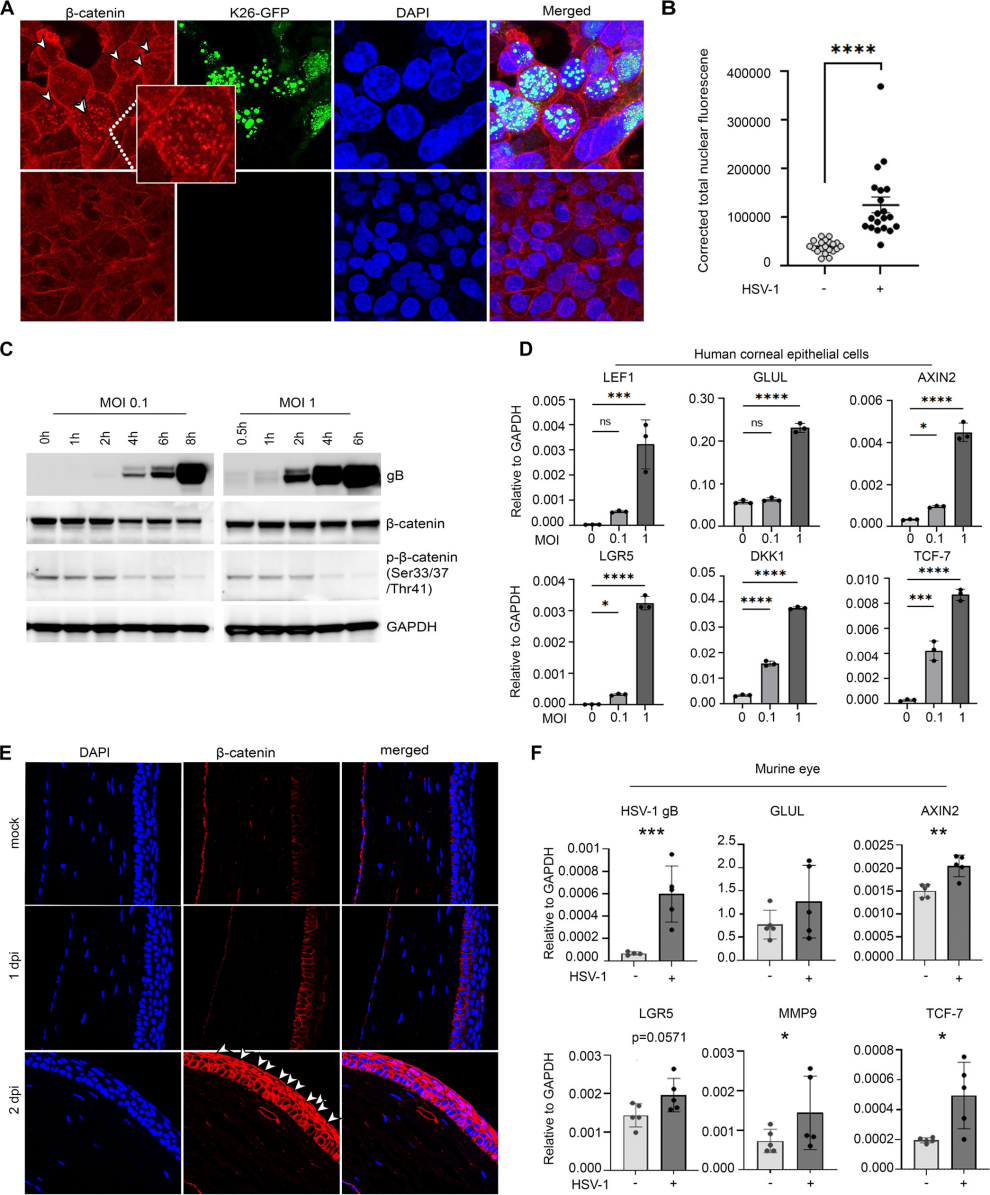
HSV-1 infection causes β-catenin nuclear translocation and signal transduction. (A) Confocal immunofluorescence microscopy of HCE cells showing β-catenin nuclear localization following infection with K26 GFP-HSV-1 at MOI of 0.1. (B) Significance between the corrected total nuclear fluorescence determined by an unpaired *t* test. (C) Western blot analysis of HSV-1 glycoprotein B (gB), β-catenin, p-β-catenin (Ser33/37/Thr41), and GAPDH at indicated time points after infection of HCE cells with HSV-1 (KOS strain). The uninfected control is indicated as the zero hour postinfection sample. (D) mRNA expression, relative to GAPDH, of key genes involved in β-catenin signal transduction following infection of HCE cells with HSV-1 (KOS strain) at MOIs of 0.1 and 1. Significance determined by one-way analysis of variance (ANOVA) with Dunnett’s multiple-comparison test. (E) Confocal immunofluorescence microscopy of mouse corneal sections probing for β-catenin protein expression and localization following 1 and 2 days postinfection with HSV-1 (McKrae strain) at 4 × 10^5^ PFU/ml. Arrows indicate cells with apparent β-catenin nuclear localization in the corneal epithelium. (F) mRNA expression, relative to β-actin, of key genes involved in β-catenin signal transduction in the mouse eye at 2 dpi with HSV-1 (McKrae strain) at 4 × 10^5^ PFU/ml. Significance between the corrected total nuclear fluorescence determined by an unpaired *t* test. For all statistical analyses, *, *P* < 0.05; **, *P* < 0.01; ***, *P* < 0.001; and ****, *P* < 0.0001.

Recent studies have highlighted the dysregulation of Wnt/β-catenin signaling pathway due to viral replication (20–23). However, the current findings are not in line with one another and therefore require more robust understanding of the role of β-catenin in HSV-1 replication. Here, we use both laboratory and clinical strains of HSV-1 and use the eye as a clinically relevant model of infection. We initially probed for the localization of β-catenin in the absence and presence of HSV-1 infection using human corneal epithelial (HCE) cells. HCE cells were infected with HSV-1 KOS strain for 24 h. The cells were then fixed and immunostained for β-catenin. Imaging with confocal microscopy indicated that, under the mock-infected condition, β-catenin was localized predominantly along the adherens junctions. Following HSV-1 infection and replication, robust and significant nuclear localization of β-catenin was observed (Fig. 1A and B). To understand at what point after infection β-catenin is activated, we performed a time course of infection experiment at a multiplicity of infection (MOI) of both 0.1 and 1. The collected lysates were subjected to Western blot analysis and immunoblotted for HSV-1 glycoprotein B (gB), β-catenin, and p-β-catenin (Ser33/37/Thr41). Over the course of infection, β-catenin phosphorylation at Ser33/37/Thr41 decreases, suggesting that it is no longer being targeted for degradation (Fig. 1C). These findings were also confirmed using the same viral strain in human embryonic kidney (HEK) cells and MEFs (Fig. S1A and B in the supplemental material). Furthermore, the reduction of p-β-catenin (Ser33/37/Thr41) was accompanied by increased levels of phosphorylation of GSK-3β, a negative regulator of β-catenin, at Ser9, which confers GSK-3β to inhibition. To understand the clinical relevance of the dysregulation of this pathway during ocular HSV-1 infection, we used clinical isolates collected from the ocular surface of patients presenting herpes keratitis (24). Similar to results found using our laboratory strain, *in vitro* infection with the clinical isolates resulted in increased protein levels of p-GSK-3β (Ser9) and p-β-catenin (Ser33/37/Thr41) over the time course of infection (Fig. S1A to C). Furthermore, quantitative PCR (qPCR) confirmed upregulation of well-established β-catenin downstream transcriptional targets with increased MOIs in HCE cells (Fig. 1D).

10.1128/mBio.02792-21.1 FIG S1 β-catenin is activated following infection with laboratory or clinical strains of HSV-1 in multiple cell types. (A and B) Western blot analysis of HEK293 cells (A) and MEFs (B) infected with of HSV-1 KOS strain at MOI of 0.1 and collected at the indicated time points. (C) Western blot analysis of HCE cells infected with indicated time points of HSV-1 clinical strain U or B at MOI of 0.1. (D) Confocal immunofluorescence microscopy of HSV-1 in mouse corneal sections following 1 and 2 dpi with HSV-1 McKrae strain at 4 × 10^5^ PFU/ml. Download FIG S1, PDF file, 1.2 MB. Copyright © 2021 Koujah et al. 2021 Koujah et al. https://creativecommons.org/licenses/by/4.0/ This content is distributed under the terms of the Creative Commons Attribution 4.0 International license.

After verifying activation and nuclear translocation of β-catenin *in vitro*, we sought to confirm these findings *in vivo*. C57BL/6 mice were infected with 4 × 10^5^ PFU of HSV-1 McKrae strain, and eyes were collected and fixed at 1 and 2 days postinfection (dpi). Starting at 1 dpi, upregulation of β-catenin protein was observed compared to mock-infected corneas. By 2 dpi, significant upregulation of β-catenin was observed, along with nuclear translocation in the superficial-most layer of the corneal epithelium, where tight and adherens junctions are present to provide a permeability barrier between cells (Fig. 1E). HSV-1 infection likely causes disruption of these junction barriers, leading to the subsequent release of β-catenin that is bound to the intracellular domain of E-cadherin. β-Catenin nuclear localization correlated with increased viral protein production in the corneal epithelium (Fig. S1D). To confirm β-catenin signal transduction is occurring, whole eyes of mice infected with 4 × 10^5^ PFU of HSV-1 McKrae were processed and subjected to qPCR. At 2 dpi, viral transcripts were detected, as well as upregulation of β-catenin downstream targets (Fig. 1F).

### HPSE promotes β-catenin activation and signal transduction following HSV-1 replication.

**FIG 2:**
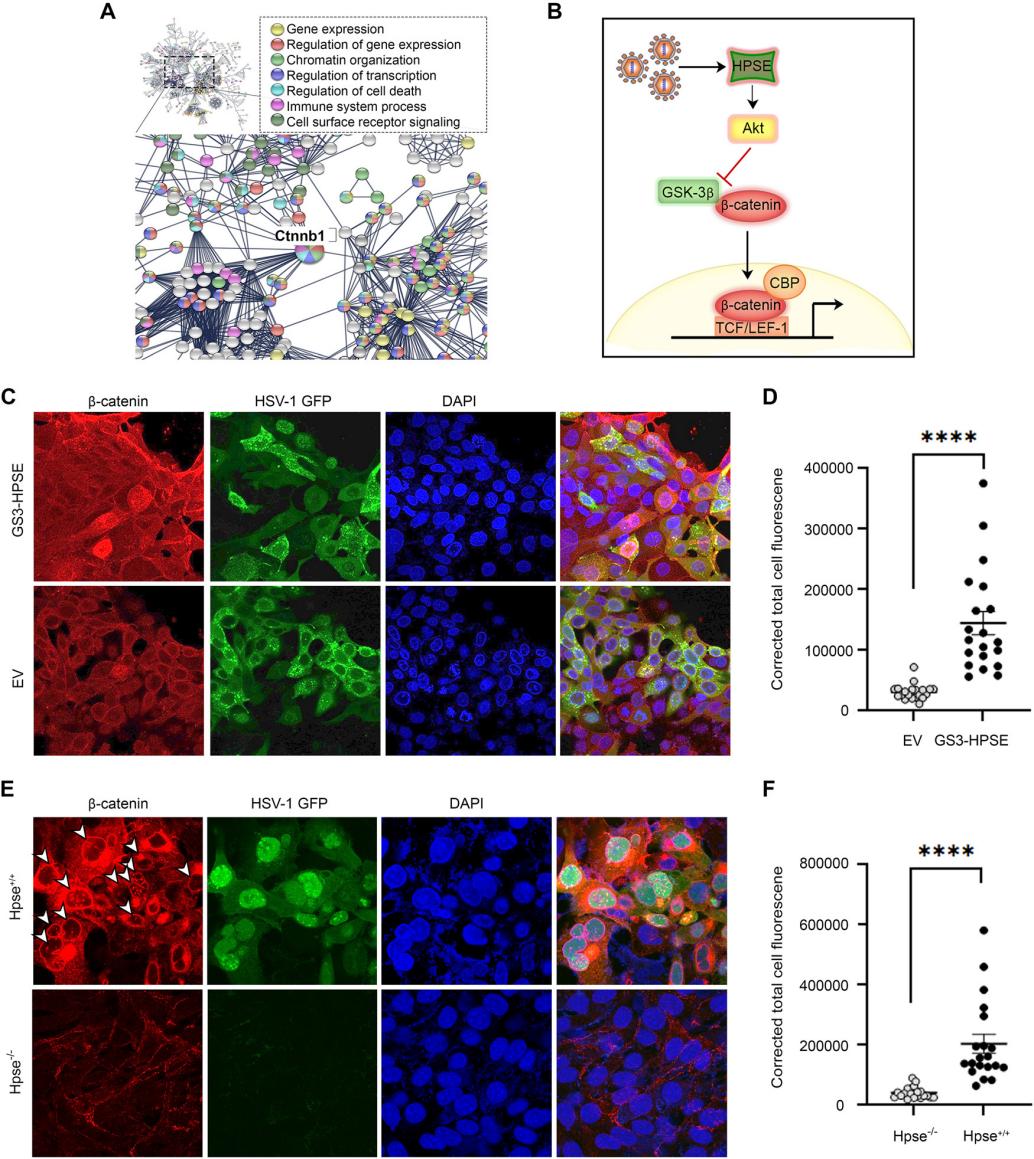
HPSE contributes to β-catenin activation and signal transduction following HSV-1 infection. (A) Rationale for mechanistic investigation of role of β-catenin in HSV-1 infection. Using quantitative proteomics analysis of HSV-1 infection time course of wild-type and Hpse-KO MEFs, β-catenin (Ctnnb1) was identified as a major differentially expression hub gene. (B) Proposed mechanism for HPSE involvement in β-catenin signaling following HSV-1 infection. (C) Confocal immunofluorescence microscopy of β-catenin expression and localization in HCE cells transfected with constitutively active HPSE (GS3-HPSE) or empty vector (EV) and infected with HSV-1 K26-GFP at MOI of 0.1. (D) Significance between the corrected total nuclear fluorescence determined by an unpaired *t* test. (E) Confocal immunofluorescence microscopy of β-catenin expression and localization in WT and Hpse-KO MEFs infected with HSV-1 K26-GFP at MOI of 0.1. Arrows indicate cells where β-catenin expression is heightened and nuclear localization is observed. (F) Significance between the corrected total nuclear fluorescence determined by an unpaired *t* test. For all statistical analysis, *, *P* < 0.05; **, *P* < 0.01; ***, *P* < 0.001; and ****, *P* < 0.0001.

Using quantitative proteomics analysis of HSV-1 infection time course of wild-type and Hpse-KO MEFs, β-catenin was identified as a major differentially expressed hub gene (Fig. 2A) (5). This prompted further investigation regarding the relationship between the two proteins. Specifically, we sought to exam whether virus-induced upregulation of HPSE contributed to the propagation of β-catenin signal transduction through the inhibition of Akt and subsequently GSK-3β, a negative regulator of β-catenin. Having established that HSV-1 replication causes dysregulation in the β-catenin signal transduction pathway, we proceeded to characterize the relationship between β-catenin and HPSE during infectious conditions. We found that HCE cells expressing constitutively active HPSE (GS3-HPSE) exhibited increased nuclear localization of β-catenin, suggesting an important role for HPSE in β-catenin signal transduction. This was then supported by the observation that cells lacking HPSE exhibited lower endogenous protein levels of β-catenin and that following infection and replication, β-catenin nuclear translocation was not observed in cells lacking HPSE (Fig. S2; Fig. 2E and F). These studies confirm the interdependence between β-catenin and HPSE during HSV-1 replication.

10.1128/mBio.02792-21.2 FIG S2 Hpse-KO cells have endogenously lower levels of β-catenin protein expression. (A) Confocal immunofluorescence microscopy of WT and Hpse-KO MEFs stained for β-catenin. (B) Confocal immunofluorescence microscopy of β-catenin in Hpse-KO mouse corneal sections following 1 and 2 dpi with HSV-1 McKrae strain at 4 × 10^5^ PFU/ml. Download FIG S2, PDF file, 2.1 MB. Copyright © 2021 Koujah et al. 2021 Koujah et al. https://creativecommons.org/licenses/by/4.0/ This content is distributed under the terms of the Creative Commons Attribution 4.0 International license.

### Virus-induced upregulation of HPSE leads to the phosphorylation of GSK-3β and enhancement of viral replication.

**FIG 3:**
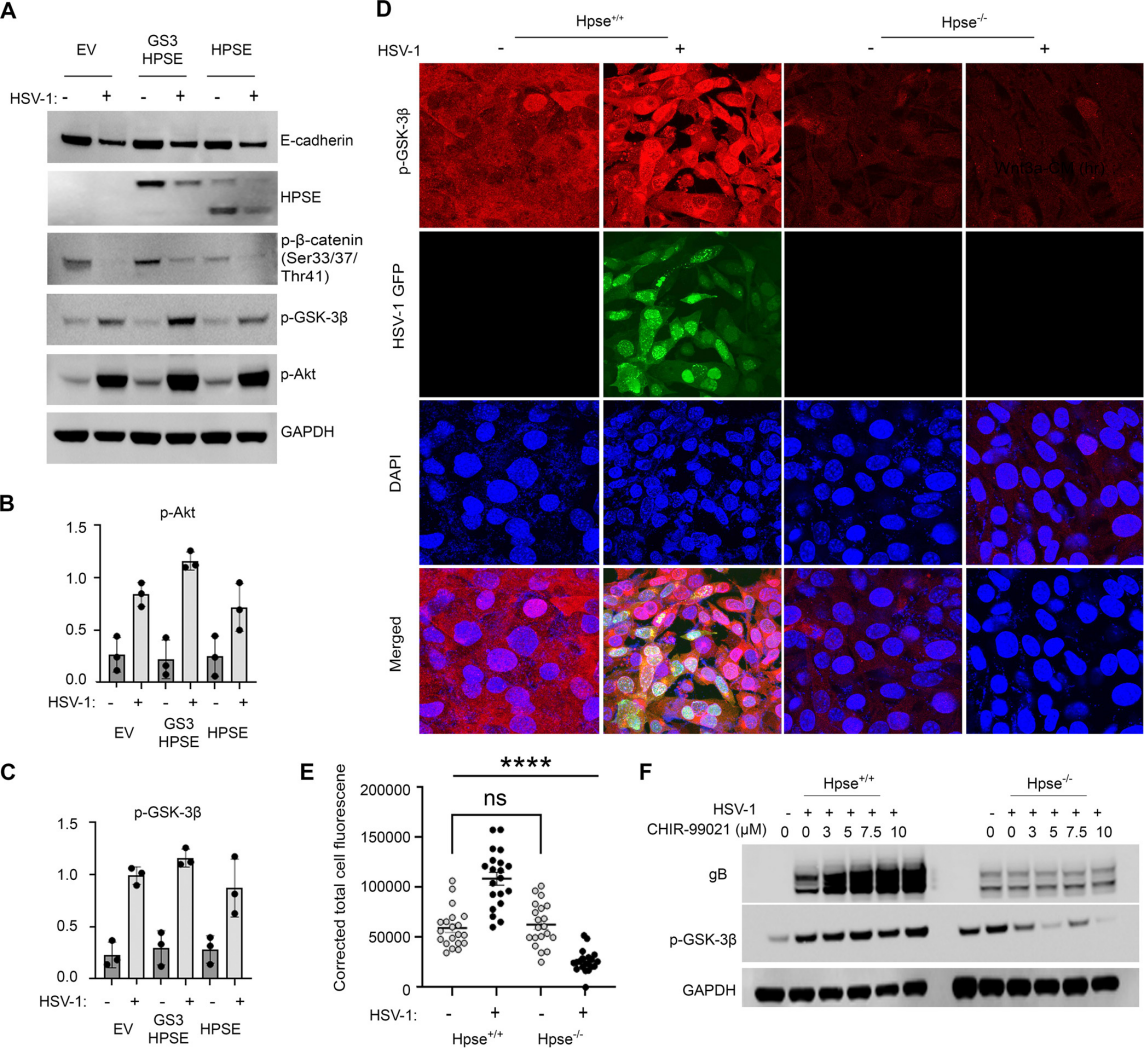
Virus-induced upregulation of HPSE leads to the phosphorylation of GSK-3β and enhancement of viral replication. (A) Representative Western blot analysis of key proteins involved in regulation of β-catenin. HCE cells were transfected with GS3-HPSE or latent HPSE and infected with HSV-1 (KOS strain) at an MOI of 0.1 for 24 h. (B and C) Quantification of p-Akt (B) and p-GSK-3β (C) expression. (D) Confocal immunofluorescence microscopy of p-GSK-3β in WT and Hpse-KO MEFs infected with HSV-1 K26-GFP at MOI of 0.1. (E) Significance between the corrected total nuclear fluorescence determined by one-way ANOVA with Tukey’s multiple-comparison test. *, *P* < 0.05; **, *P* < 0.01; ***, *P* < 0.001; ****, *P* < 0.0001; ns, not significant. (F) Western blot analysis of WT and Hpse-KO MEF cells treated with increasing concentrations of CHIR-99021 for 6 h prior to infecting with HSV-1 (KOS strain) at MOI of 0.1 for 24 h.

The HPSE gene encodes a proenzyme whose activation involved proteolytic cleavage by cathepsin L (CatL), yielding an enzymatically active heterodimer (4). To understand the differential contributions of active and proenzyme forms of HPSE on the phosphorylation of Akt and GSK-3β leading to the subsequent activation of β-catenin, HCE cells were transfected with plasmids expressing constitutively active HPSE (GS3-HPSE) or proenzyme HPSE. These cells were infected with HSV-1 KOS strain at an MOI of 0.1 and subjected to Western blot analysis (Fig. 3A). HCE cells expressing GS3-HPSE exhibited significantly greater levels of phosphorylation of Akt and GSK-3β than cells transfected with empty vector (EV) or proenzyme HPSE in the presence of infection (Fig. 3B and C). β-Catenin is bound to the intracellular domain of E-cadherin, and disruption of the adherens junctions results in the release of β-catenin (25). HPSE dysregulation is also involved in extracellular matrix remodeling and disruption of the adherens junction (26). Immunoblotting of E-cadherin was also performed to assess whether changes to the total protein levels were made as a result of infection or upregulation of HPSE and whether this contributes to the potentiation of β-catenin. While HSV-1 infection does result in a decrease of E-cadherin protein levels, the amount reduced remained constant in the presence of GS3-HPSE or HPSE upregulation (Fig. 1A).

After observing the direct role of HPSE on regulating β-catenin activity through phosphorylation of upstream targets, we sought to verify these findings by examining the phosphorylation of GSK-3β in wild-type and Hpse-KO MEFs. Following infection with a KOS HSV-1 strain with an MOI of 0.1, a significant increase in phosphorylation of GSK-3β was observed in the wild-type cells. However, the opposite trend was observed in HPSE knockout cells where the expression level of p-GSK-3β (Ser9) was reduced following infection (Fig. 3D). In order to establish a state where β-catenin was already active prior to infection, CHIR-99021, a potent GSK-3 inhibitor, was used to treat cells prior to infection. To confirm the effects of this drug, HCE cells were treated with 10 μM CHIR-99021 for 6 h prior to HSV-1 infection. Confocal microscopy confirms enhanced nuclear localization of β-catenin in cells pretreated with CHIR-99021 (Fig. S3A). Furthermore, treatment of wild-type MEFs with increasing concentrations of CHIR-99021 resulted in a dose-dependent increase of viral protein production in concert with increased phosphorylation of GSK-3β. However, CHIR-99021 had no effect on viral production in Hpse-KO cells. While we had expected no changes to be observed in the phosphorylation of GSK-3β in the absence of HPSE, the levels decreased while the concentration of CHIR-99021 increased (Fig. 3F).

10.1128/mBio.02792-21.3 FIG S3 CHIR-99021 enhances β-catenin nuclear localization. (A) Confocal immunofluorescence microscopy of cells treated with CHIR-99021 for 24 h prior to HSV-1 infection with MOI of 0.1 of HSV-1 infection in HCE cells. Download FIG S3, PDF file, 1.9 MB. Copyright © 2021 Koujah et al. 2021 Koujah et al. https://creativecommons.org/licenses/by/4.0/ This content is distributed under the terms of the Creative Commons Attribution 4.0 International license.

### Absence of HPSE renders cells resistant to phosphorylation of β-catenin at serine 552.

**FIG 4:**
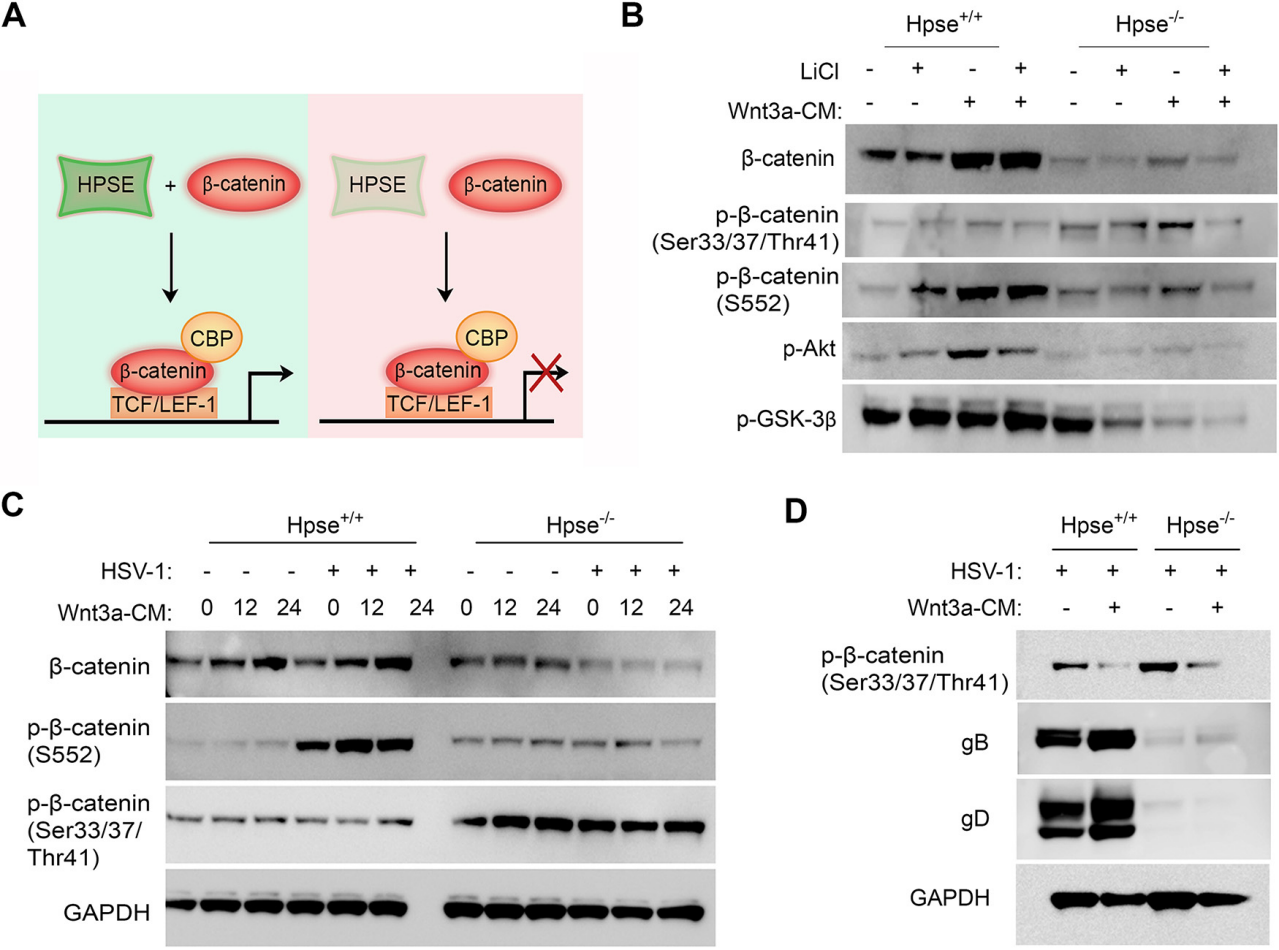
Absence of HPSE renders cells resistant to phosphorylation of β-catenin at serine 552. (A) General schematic of the observed findings regarding the role of HPSE on β-catenin signaling. (B) Western blot analysis of WT and Hpse-KO MEF cells treated with 20 μM of LiCl, Wnt3a-CM, or a combination of both for 24 h. (C) Western blot analysis of WT and Hpse-KO MEF cells treated with Wnt3a-CM for the indicated time prior to infection with HSV-1 (KOS strain) at MOI of 0.1 for 24 h. (D) Western blot analysis of viral glycoproteins in WT and Hpse-KO MEF cells treated with Wnt3a-CM for 24 h prior to infection with HSV-1 (KOS strain) at MOI of 0.1 for 24 h.

After establishing a key role for HPSE in phosphorylation of upstream targets of β-catenin, Akt, and GSK-3β, we next explored the downstream repercussions of HPSE activity on phosphorylation of β-catenin at Ser552. Previous findings have indicated that phosphorylation of β-catenin at Ser552 by Akt promotes β-catenin transcriptional activity. Prior reports have also shown that HPSE is involved in the induction of phosphorylation of Akt. To investigate this relationship further, wild-type and Hpse-KO cells were treated for 24 h with independent or combinatorial administration of lithium chloride (LiCl), an inhibitor of GSK-3β, or Wnt3a-conditioned medium (Wnt3a-CM) (Fig. 4B). As expected, in wild-type cells, treatment with LiCl increased expression of p-GSK-3β (Ser9) and subsequently increased p-β-catenin (S552). Treatment of the wild-type cells with Wnt3a-CM resulted in increased β-catenin, p-β-catenin (S552), and p-Akt protein levels. Furthermore, the heightened protein expression was also observed following combinatorial administration of LiCl and Wnt3a-CM. However, treatment with either LiCl or Wnt3a-CM did not cause any changes in p-Akt, p-β-catenin (S552), or total β-catenin protein levels in Hpse-KO MEFs. Similar to previous findings, inhibition of GSK-3β or induction of β-catenin signaling pathway results in reduction of, rather than increase of, p-GSK-3β (Ser9) in the absence of HPSE. These findings suggest that HPSE is required for phosphorylation of β-catenin at S552 and for the subsequent activation of β-catenin.

To further understand the consequences of HPSE regulation of β-catenin activation on HSV-1 infection, wild-type and Hpse-KO cells were primed with Wnt3a-CM for 12 or 24 h prior to infection with a KOS strain with an MOI of 0.1 for 24 h (Fig. 4C). Treatment with Wnt3a-CM resulted in the accumulation and activation of β-catenin only in the presence of HPSE. Furthermore, promoting β-catenin activation prior to infection resulted in enhanced viral protein production, indicating that β-catenin plays a proviral role during HSV-1 infection (Fig. 4D).

### β-Catenin promotes HSV-1 replication.

**FIG 5:**
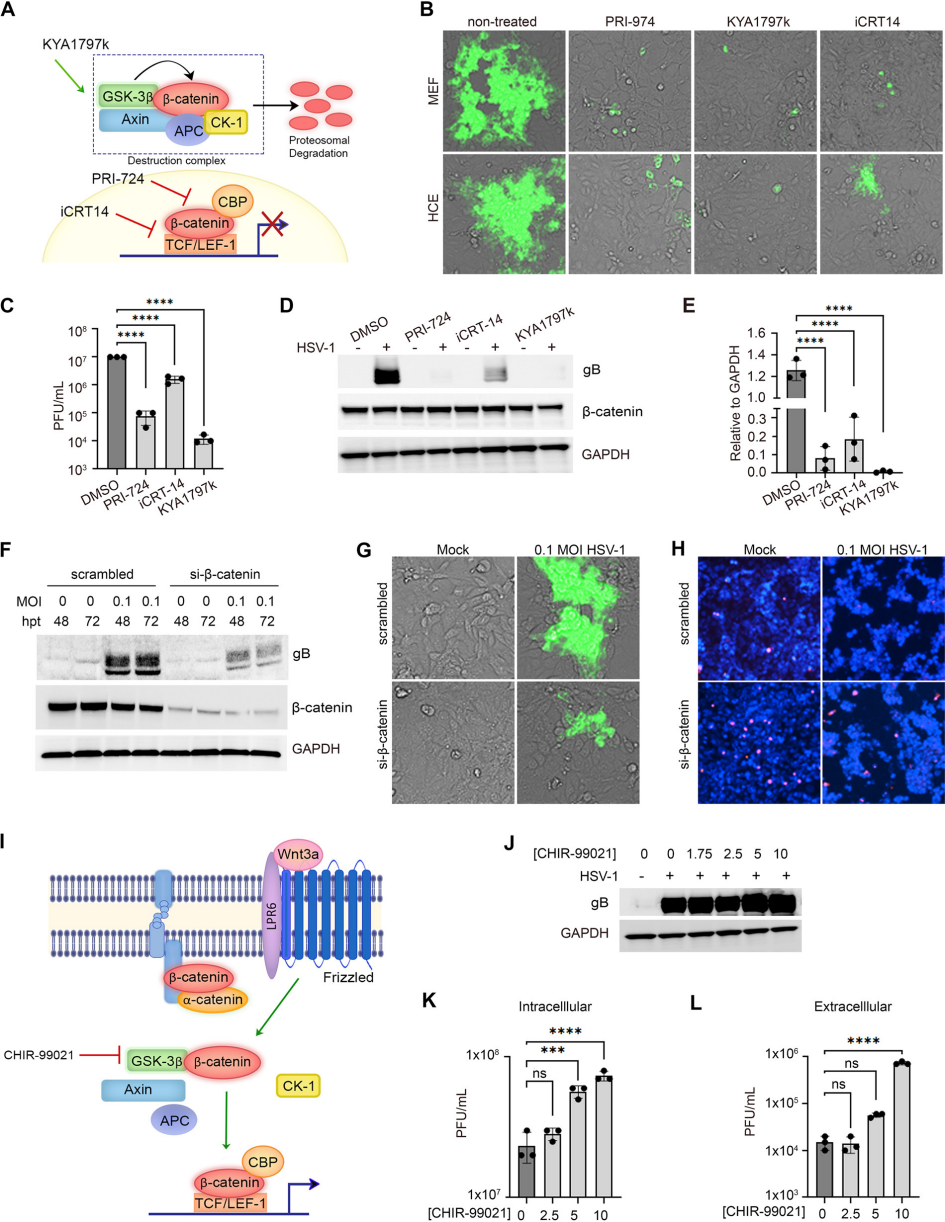
β-Catenin promotes HSV-1 replication. (A) Diagram indicating the targets of each drug used in the study. Green arrow represents the activation of, and red bar indicates inhibition of, respective protein or interaction. (B) Immunofluorescence microscopy of HCE and MEF cells infected with HSV-1 K26-GFP at MOI of 0.1 and therapeutically treated with 0.1% DMSO, PRI-974 (50 μM), KYA1797k (40 μM), or iCRT14 (10 μM). (C) Infectious particles were assessed by plaque assay. (D) Representative Western blot analysis of total β-catenin protein levels and HSV-1 gB following therapeutic treatment with β-catenin inhibitors in HCE cells. (E) Quantification of Western blot analysis of HSV-1 gB relative to GAPDH. (F) Western blot analysis of viral protein production in HCE cells lacking β-catenin protein expression. Cells were transfected with si-β-catenin for 48 or 72 h prior to infection with HSV-1 KOS strain at MOI of 0.1 for 24 h. (G) Immunofluorescence microscopy of HCE cells infected with HSV-1 K26-GFP at MOI of 0.1 for 24 h after transfection with scrambled control or si-β-catenin for 48 h. (H) Propidium iodide staining of the cells in panel G. (I) Diagram indicating the mechanism of action of CHIR-99021. (J) Western blot analysis of HCE cells treated with various concentrations of CHIR-99021 for 24 h prior to infection with HSV-1 KOS strain at MOI of 0.1. (K and L) Intracellular (K) and extracellular (L) infectious particles collected from HCE cells treated with various concentrations of CHIR-99021 prior to infection were assessed by plaque assay. Statistical significance was determined by one-way ANOVA with Dunnett’s multiple-comparison test. *, *P* < 0.05; **, *P* < 0.01; ***, *P* < 0.001; ****, *P* < 0.0001.

Given the ambiguity in the field, we sought to further understand the role that β-catenin plays during viral infection in our model of infection. Potent β-catenin inhibitors were used to interrogate the pathway and further probe for the effect of β-catenin on HSV-1 replication. KYA1797k, a drug that destabilizes β-catenin by promoting β-catenin degradation, PRI-724, an inhibitor of β-catenin binding to transcriptional coactivator, and iCRT14, a potent inhibitor of β-catenin binding to transcriptional coactivator TCF/LEF-1, all displayed robust and significant suppression of viral replication and protein production in HCE cells and MEFs (Fig. 5A and B). The effect of these inhibitors of β-catenin was also observed in a dose-dependent manner to determine the effective concentration for use (Fig. S4A to D). Bright-field images shown indicate no decrease in cell viability when any of the drugs are used at the therapeutic concentrations. A cell viability assay was performed for KYA1797k to further confirm this observation. Extensive data have been published regarding the toxicity of PRI-724 and iCRT14 *in vitro*, *in vivo*, and in humans and show limited adverse effects when used at the therapeutic concentrations used in this study (16, 27, 28; ClinicalTrials.gov registration nos. NCT03620474 and NCT01606579). PRI-724 and iCRT14 were further investigated against clinical strains of ocular HSV-1, and results indicate that these drugs display antiviral activity against both laboratory and clinical HSV-1 strains (Fig. S4F and G).

10.1128/mBio.02792-21.4 FIG S4 Inhibition or lack of β-catenin produces a robust antiviral state against HSV-1 infection. (A) Immunofluorescence micrographs of HCE cells infected with HSV-1 K26-GFP at MOI of 0.1 and therapeutically treated with the indicated concentration of mock (0.1% DMSO), PRI-724. or iCRT14. (B) Immunofluorescence micrographs of HCE cells infected with HSV-1 K26-RFP at MOI of 0.1 and therapeutically treated with indicated concentrations of KYA1797k. (C and D) Intracellular (C) and extracellular (D) infectious particles collected from HCE cells therapeutically treated with various concentrations of KYA1797k following infection were assessed by plaque assay. (E) Percent cell viability of cells following treatment with KYA1797k at increasing concentrations in HCE cells. (F and G) Western blot analysis of viral protein gB following therapeutic treatment with PRI-724 and iCRT14 after infection with HSV-1 laboratory KOS strain (F) and clinical strain BLS or ULS (G) in HCE cells. (H) Western blot analysis of viral protein production in HCE cells lacking β-catenin protein expression. Cells were transfected with si-β-catenin for 48 h prior to infection with HSV-1 KOS strain at MOI of 0.1 for the indicated time points. Download FIG S4, PDF file, 1.6 MB. Copyright © 2021 Koujah et al. 2021 Koujah et al. https://creativecommons.org/licenses/by/4.0/ This content is distributed under the terms of the Creative Commons Attribution 4.0 International license.

Furthermore, gene silencing of β-catenin through the use of small interfering RNA (siRNA) was used to confirm that the absence of β-catenin results in reduction of HSV-1 infection (Fig. 5F and G). The absence of β-catenin hindered viral protein production as early as 16 h postinfection (hpi), and the effect was sustained until 36 hpi in HCE cells (Fig. S4H). β-Catenin signaling is known to play a role in cell survival and proliferative pathways, and the absence of β-catenin subjects cells to cell death (29). Using a propidium iodide stain, we show that cells lacking β-catenin, while having reduced levels of HSV-1 infection, have heightened amounts of cell death (Fig. 5H).

The effect of β-catenin activation by CHIR-99021 was also assessed in HCE cells (Fig. 5I to L). CHIR-99021 is an inhibitor of GSK-3β which ultimately shields β-catenin from phosphorylation by GSK-3β and degradation. HCE cells were first treated with CHIR-99021 at multiple concentrations for 24 h prior to infection with KOS HSV-1 strain with an MOI of 0.1. We observed increased viral gB production with increased concentrations of CHIR-99021 (Fig. 5J). Furthermore, the intracellular and secreted PFU were assessed under different treatment concentrations. A significant increase of viral replication was observed at both 5 and 10 μM, and significantly increased viral secretion was observed at 10 μM concentration (Fig. 5K and L).

### HPSE potentiates β-catenin activation and signal transduction to enhance viral replication.

**FIG 6:**
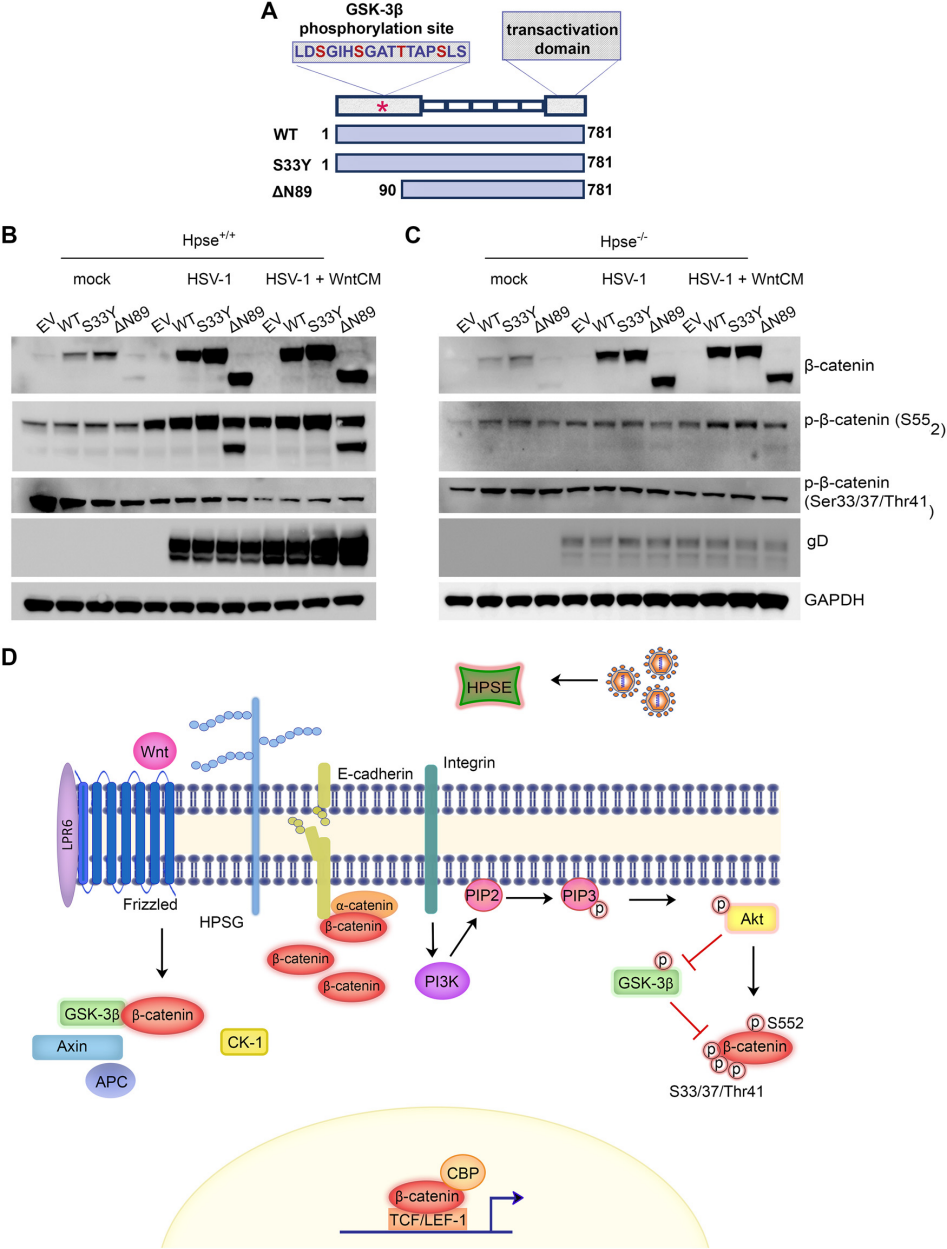
HPSE potentiates β-catenin activation and signal transduction to enhance viral replication. (A) Diagram of β-catenin plasmid constructs used with indicated mutations or truncations. (B and C) Western blot analysis of WT MEF cells (B) and Hpse-KO (C) cells transfected with control empty vector (EV), WT β-catenin, constitutively active β-catenin (S33Y), and truncated β-catenin (deltaN89). The cells were then either infected with HSV-1 or pretreated for 4 h with Wnt3a-CM prior to infection for 24 h. (D) Model of HPSE function and propagation of Akt signaling pathway leading to β-catenin stabilization and activation.

After having observed the dependence of β-catenin activation on HPSE to promote viral replication during HSV-1 infection, we sought to determine whether expression of transcriptionally active β-catenin would promote HSV infection in the absence of HPSE. Three plasmid constructs were used expressing wild-type (WT), constitutively active (S33Y), and a truncated and transcriptionally active (ΔN89) β-catenin (Fig. 6A). After transfection, the cells were infected with HSV-1 or treated for 4 h with Wnt3a-CM prior to infection for 24 h. Wnt3a-CM pretreatment was used to ensure sustained plasmid expression and activation of β-catenin signal transduction. While no changes were observed in viral protein production with overexpression of any plasmid construct, pretreatment with Wnt3a-CM prior to infection increased viral protein production under all conditions but most prominently in cells expressing the two transcriptionally active forms of β-catenin (S33Y and ΔN89) (Fig. 6B). Wnt3a-CM treatment or overexpression of any of the β-catenin constructs did not elicit increased viral replication in the absence of HPSE (Fig. 6C).

## DISCUSSION

Dysregulation of the Wnt/β-catenin pathway has been implicated in the onset of diseases, including various cancers as well as viral infections such as influenza virus, hepatitis C virus (HCV), hepatitis B virus (HBV), herpesviruses, and human immunodeficiency virus (HIV), among others (20–22, 28, 30–36). While results from these studies confirm a role for Wnt/β-catenin signaling in disruption of normal cellular function following infection, the reports diverge regarding the central role of this pathway and the mechanism by which it is regulated. Furthermore, the cell type, cellular context, and pathogen play a critical role in assessing the contributions of this pathway.

Host signaling pathways are altered during pathological conditions like HSV-1 infection, and better understanding of the vast host interactome reconfiguration will conceptually advance the field and lead to the identification of novel drug targets. In this study, we examine the role of Wnt/β-catenin pathway in physiologically relevant *in vitro* and *in vivo* contexts. We report, for the first time, β-catenin activation, nuclear translocation, and signal transduction in HCE cells and the murine cornea following HSV-1 infection. Furthermore, pharmacological inhibition of β-catenin establishes an antiviral cellular environment at therapeutically safe concentrations. We also study the mechanisms leading to β-catenin activation and show that HPSE is a key regulator of β-catenin signaling. HPSE and β-catenin have independently been implicated in regulating key processes in pathological conditions; however, the relationship between the two proteins has never been examined.

Previous studies by our lab examined the effect of virus-induced upregulation of HPSE during HSV-1 infection, which, in turn, generates a proviral environment through the cleavage of heparan sulfate proteoglycans, permitting the release of virus particles and reinfection of surrounding cells (2–4). In more recent studies, we highlighted HPSE as a multifunctional protein driving key functions, including cellular proliferation and host defense mechanisms. Specifically, HPSE regulates key cellular processes to evade innate defense responses leading to cell death and, in turn, triggers prosurvival transcription through NF-κB, Akt, CREB, and β-catenin signaling (5). In this study, we expand on our previous works and delve into the mechanism of HPSE involvement in these signaling pathways. We initially overexpressed constitutively active HPSE (GS3-HPSE) and used confocal microscopy to examine β-catenin cellular localization. GS3-HPSE expression resulted in significantly enhanced β-catenin upregulation and nuclear localization, indicating a role for HPSE in potentiating β-catenin activation. Furthermore, we observed that cells lacking HPSE have endogenously lower levels of β-catenin and that infecting these cells does not result in β-catenin nuclear localization. This warranted further investigation regarding the critical role of HPSE in β-catenin signal transduction.

Several studies have shown that Akt activation and phosphorylation of GSK-3β lead to the stabilization of β-catenin and release from the destruction complex (37). This is then followed by β-catenin nuclear localization and signal transduction. We show that HPSE regulates intracellular levels of β-catenin through Akt-mediated phosphorylation and inactivation of GSK-3β. Interestingly, cells that were primed with CHIR-99021, a drug that inhibits GSK-3β and allows for β-catenin activation, results in enhanced productive viral replication. However, in the absence of HPSE, CHIR-99021 does not have an effect on viral replication.

We next examined the differential contributions of HPSE on phosphorylation of β-catenin. The N terminus of cytosolic β-catenin is constitutively phosphorylated by a multiprotein complex commonly referred to as the destruction complex, which is bound to the scaffold protein Axin. Among other proteins, Axin has binding sites for β-catenin, GSK-3β, and casein kinase 1 (CK1). CK1 primes β-catenin by phosphorylating it at Ser45 prior to GSK-3β phosphorylating β-catenin at Ser33, Ser37, and Thr41. When β-catenin is phosphorylated at these sites, it is ultimately recognized by the β-TrCP E3 ubiquitin-ligase complex, ubiquitinylated, and rapidly degraded by the 26S proteasome. β-Catenin encompasses other phosphorylation sites that, rather than tagging it for degradation, enhance the signaling activity. Of note is the phosphorylation of β-catenin at Ser552 by Akt, which enhances β-catenin protein levels and nuclear signaling (38). Since HPSE has been implicated in indirectly phosphorylating and activating Akt, we sought to further interrogate this pathway and confirm β-catenin activation dependence on HPSE through phosphorylation at Ser552 by Akt. We initially used LiCl, a GSK-3β inhibitor, and Wnt3a-CM to establish a state where β-catenin is active or not being actively targeted for degradation. We found that in wild-type cells, treatment with either LiCl or Wnt3a-CM resulted in phosphorylation of GSK-3β, Akt, and β-catenin (Ser552). However, in the absence of HPSE, the cells were resistant to phosphorylation of Akt and β-catenin (Ser552). Interestingly, the phosphorylation of GSK-3β was suppressed with these treatments rather than enhanced. This was similar to the observation that treatment with CHIR-99021 increased p-GSK-3β in wild-type cells but decreased the levels of p-GSK-3β in the absence of HPSE. These results indicate that GSK-3β is also differentially regulated in the presence and absence of HPSE, although the mechanism by which this occurs is currently unknown.

We next sought to investigate the levels of β-catenin phosphorylation in response to HSV-1 infection in the presence and absence of HPSE. We pretreated the cells with Wnt3a-CM prior to infection to prevent the degradation of β-catenin. As expected, in the wild-type cells, treatment with Wnt3a-CM prior to infecting caused a robust increase in p-β-catenin (Ser552) only in the presence of HPSE. This increase in active β-catenin caused an increase in viral protein production, further proving the proviral role of β-catenin. Furthermore, introduction of β-catenin plasmids expressing wild-type constitutively active S33Y mutation and constitutively active deltaN89 truncation showed that, following Wnt3a-CM treatment and HSV-1 infection, expression of constitutively active β-catenin resulted in enhanced viral protein production in wild-type cells. This correlated with increased protein expression of p-β-catenin Ser552 and a decrease in p-β-catenin S33/S37/Thr41. However, in the absence of HPSE, expression of β-catenin plasmids did not result in any changes to HSV-1 infection, and phosphorylation of β-catenin remained constant under all conditions. These studies confirm that β-catenin activation is dependent on a kinase cascade beginning with HPSE-induced phosphorylation of Akt, leading to the phosphorylation of β-catenin at S552.

In summary, our findings assign a novel regulatory role to HPSE as a driver of β-catenin signaling during HSV-1 infection. While further investigation is required to determine the mechanism of HPSE-induced Akt activation, this study provides key insights on the cross talk of cell signaling pathways that are conducive to pathogenic milieu and viral replication. Our findings also support further development of therapeutic agents targeting HPSE and/or β-catenin signaling pathway to combat herpesvirus infection and, quite possibly, many other pathological conditions.

## MATERIALS AND METHODS

### Western blotting.

Cellular proteins were extracted using radioimmunoprecipitation (RIPA; Sigma) buffer and Halt protease inhibitor cocktail (Thermo Scientific). Lysis was performed on ice with agitation for 1 h, followed by 10 min centrifugation at 13,000 rpm. After removing cellular debris, lysates were then denatured at 95°C for 8 min in the presence of 4× LDS sample loading buffer (Life Technologies) and 5% β-mercaptoethanol (Bio-Rad, Hercules, CA) and electrophoresed by SDS-PAGE with NuPAGE 4 to 12% Bis-Tris 1.5-mm 15-well gels (Thermo Scientific). Proteins were then transferred to nitrocellulose using iBlot2 system (Thermo Scientific), and membranes were blocked in 5% milk/Tris-buffered saline with Tween 20 (TBST) for 1 h at room temperature, followed by incubation with primary antibody overnight at 4°C. After washes with TBST and incubation with respective horseradish peroxidase-conjugated secondary antibodies for 1 h at room temperature, protein bands were visualized using SuperSignal West Femto substrate (Thermo Scientific) with Image-Quant LAS 4000 biomolecular imager (GE Healthcare Life Sciences, Pittsburgh, PA).

### Antibodies, plasmids, and siRNA.

For Western blotting, the housekeeping gene GAPDH (glyceraldehyde-3-phosphate dehydrogenase) was used for normalization as a loading control. Antibodies used in this study are GAPDH (Proteintech; catalog no. 10494) at dilution 1:1,000, gB (Abcam; catalog no. ab6505) at dilution 1:10,000, gD (Abcam; catalog no. ab6507) at dilution 1:10,000, β-catenin (CST Signaling; catalog no. D10A8) at dilution 1:1,000, phospho-β-catenin (Ser33/37/Thr41) (CST Signaling; catalog no. 9561) at dilution 1:1,000, phospho-β-catenin (Ser552) (CST Signaling; catalog no. D8E11) at dilution 1:1,000, phospho-GSK-3β (Santa Cruz Biotechnology; catalog no. SC-373800) at dilution 1:1,000, GSK-3β (Santa Cruz Biotechnology; catalog no. SC-377213) at dilution 1:1,000, E-cadherin (Santa Cruz Biotechnology; catalog no. SC-8426) at dilution 1:1,000, HPSE (Abcam; catalog no. ab85543) at dilution 1:1,000, and phospho-Akt (Ser473) (CST Signaling; catalog no. 9271) at dilution 1:1,000. HPSE expression constructs, including the Myc-GS3 plasmid, were provided by Israel Vlodavsky (Rappaport Institute, Haifa, Israel). The following plasmids were created by Eric Fearon lab and acquired through Addgene: human β-catenin pcDNA3 (plasmid no. 16828), pcDNA3 deltaN89 β-catenin (plasmid no. 19288), and pcDNA3-S33Y β-catenin (plasmid no. 19286). Lipofectamine 2000 transfection reagent (Invitrogen; catalog no. 11668019) was used for all *in vitro* overexpression experiments according to the manufacturer’s specifications. The siRNA of β-catenin was obtained from Dharmacon (catalog nos. D-003482-01-0002 and D-003482-02-0002).

### Quantitative PCR.

RNA was extracted from cultured cells using TRIzol (Thermo Scientific; catalog no. 15596018) following the manufacturer’s protocol, and cDNA was produced using high-capacity cDNA reverse transcription kit (Life Technologies). For *in vivo* experiments, mouse tissue was extracted and incubated in 50 μl of 2 mg/ml collagenase D (Sigma; catalog no. C0130) in phosphate-buffered saline (PBS) for 1 h at 37°C. Tissues were then triturated with a pipette tip and resuspended in TRIzol, and extraction of RNA and cDNA was performed as described above. Real-time quantitative PCR (qPCR) was performed using Fast SYBR green master mix (Life Technologies) on QuantStudio 7 Flex system (Life Technologies).

### Immunofluorescence microscopy.

HCE cells or WT and Hpse-KO MEFs were cultured in glass-bottom dishes (MatTek Corporation). Cells were fixed in 4% paraformaldehyde for 10 min and permeabilized with 0.1% Triton-X (Thermo Fisher Scientific; catalog no. BP151) for 10 min at room temperature for intracellular labeling. This was followed by incubation with primary antibody for 1 h at room temperature. When a secondary antibody was needed, cells were incubated with respective fluorescein isothiocyanate (FITC)- or Alexa Fluor 647-conjugated secondary antibody (MilliporeSigma [catalog no. F9137] or Thermo Fisher Scientific [catalog no. A21244], respectively) at a dilution of 1:100 for 1 h at room temperature. NucBlue Live ReadyProbes Hoechst stain (Thermo Fisher Scientific; catalog no. R37605) was included with secondary antibody stains when applicable, according to manufacturer specifications.

### Murine ocular infection model.

Anesthetized mouse corneas were scarified in a 3 by 3 grid using a 30-gauge needle and infected as previously described (24).

### Immunohistochemical staining.

Collected mouse tissue was first fixed with 4% paraformaldehyde (PFA) for 24 h and then incubated in 70% ethanol. Sample processing and paraffin embedding was performed by the University of Illinois at Chicago core facilities. We took 10-μm section thickness using Microm HM 340. Following deparafinization, antigen retrieval was performed by incubating the slides in subboiling temperatures of 10 mM sodium citrate buffer with 0.05% Tween 20 at pH 6.0 for 15 min. Slides were then blocked in 3% bovine serum albumin (BSA) in TBST for 1 h at room temperature. Primary antibody was incubated overnight at 4°C using 1:100 concentration of β-catenin (CST Signaling; catalog no. D10A8). Slides were then washed for 15 min prior to incubation with Alexa Fluor 647-labeled anti-rabbit secondary antibody and DAPI (4′,6-diamidino-2-phenylindole) to label nuclei for 1 h at room temperature. ProLong Gold antifade mountant with DAPI was used to mount the slides.

### Cell lines and virus strains.

The HCE cell line was obtained from Kozaburo Hayashi (National Eye Institute, Bethesda, MD), and the cells were cultured in minimum essential media (MEM; Thermo Fisher Scientific) supplemented with 1% penicillin-streptomycin (Thermo Fisher Scientific) and 10% fetal bovine serum (FBS; Sigma-Aldrich). African green monkey fetal kidney epithelial (Vero) cells were provided from P. Spear (Northwestern University) and cultured in Dulbecco’s modified Eagle medium (DMEM; Life Technologies) with 10% FBS and 1% penicillin-streptomycin. Wild-type and Hpse-knockout mouse embryonic fibroblasts were provided by Israel Vlodavsky (Rappaport Institute). Virus strains used in these studies, HSV-1 (KOS-WT), green fluorescent protein (GFP)-HSV-1 (K26-GFP) with KOS strain background, and McKrae were provided by Patricia G. Spear (Northwestern University, Chicago, IL, USA). Acquisition of clinical strains used in this study is as previously described. All virus stocks were propagated in Vero cells and stored at −80°C. Wnt-3A-expressing cells were acquired from ATCC (CRL-2647) to culture Wnt3A-conditioned media according to the manufacturer’s protocol.

### Chemical reagents.

Pharmacological inhibitors of the β-catenin system were purchased from Selleckchem (PRI-724, catalog no. S8262, and KYA1797K, catalog no. S8327) and Tocris (iCRT14; catalog no. 4299). GSK-3 inhibitors were purchased from Selleckchem (CHIR-99021; catalog no. S1263) and Sigma-Aldrich (lithium chloride; catalog no. 203637-10G).

### Plaque assay.

Plaque assay was performed with tear samples from infected mice as well as with cell supernatant from *in vitro* experiments. Vero cell monolayers were inoculated for 2 h with virus-containing sample and incubated at 37°C and 5% CO_2_. The viral suspension was aspirated and replaced with complete DMEM containing 0.5% methyl cellulose (Fisher Scientific) for 48 to 72 h. Cells were then fixed with 100% methanol and finally stained with crystal violet solution to visualize plaques.

### Propidium iodide cell death assay.

Propidium iodide (PI; 2 mg/ml) and Hoechst nucleic acid stain were added to complete DMEM for 30 min prior to imaging. The Biotek Lionheart FX system was used to detect fluorescence intensity on PI, DAPI, and bright-field channels.

### MTT cytotoxicity assay.

Cellular viability was analyzed following treatment with β-catenin inhibitors. Briefly, HCEs were plated in a 96-well plate at a density of 1 × 10^4^ per well and left overnight until they were 80% confluent. Compounds to be tested and dimethyl sulfoxide (DMSO; control) were added to the designated wells and incubated for a period of 24 h at 37°C and 5% CO_2_. Postincubation, wells were washed with PBS twice before 100 μl of 0.5 mg/ml MTT [3-(4,5-dimethylthiazol-2-yl)-2,5-diphenyltetrazolium bromide] was added to each well and incubated for 4 h. Formazan crystals formed due to the mitochondrial activity were dissolved using acidified isopropanol (0.1% Tris-HCl in isopropanol) and transferred to a new 96-well plate. Genesis Pro Plate reader at 562 nm was used to analyze samples. Experiments were conducted in triplicates and individually repeated 5 times to compensate for pipetting errors.

### Data acquisition and statistical analysis.

GraphPad Prism software was used for statistical analysis, with bar heights representing the mean and error bars representing standard deviation. Statistical analysis in each figure is described in the legend, as well as explanations of dot plot representation. For images requiring measurement of total cell fluorescence, ImageJ was used. Briefly, the drawing/selection tool was used to select the cell of interest. From the analyze menu, “set measurements” was used to acquire area-integrated intensity and mean gray value. Next, “measure” was selected from the analyze menu where values indicating area, mean, and integrated density of fluorescence are given. An area with no fluorescence was used as background for the calculation of corrected total cell fluorescence (CTCF). The calculation used is as follows: CTCF = integrated density − (area of selected cell × mean fluorescence of background readings).

### Study approval.

All animal care and procedures were performed in accordance with the institutional and NIH guidelines and were approved by the Animal Care Committee at University of Illinois at Chicago (ACC protocol nos. 17-077 and 20-065).
