# Supplementary figures and images for: Heparanase-Induced Activation of AKT Stabilizes β-Catenin and Modulates Wnt/β-Catenin Signaling during Herpes Simplex Virus 1 Infection
Source: mBio. 2021 Nov 9;12(6):e02792-21. doi: 10.1128/mBio.02792-21 (PMC8576534; doi:10.1128/mBio.02792-21)

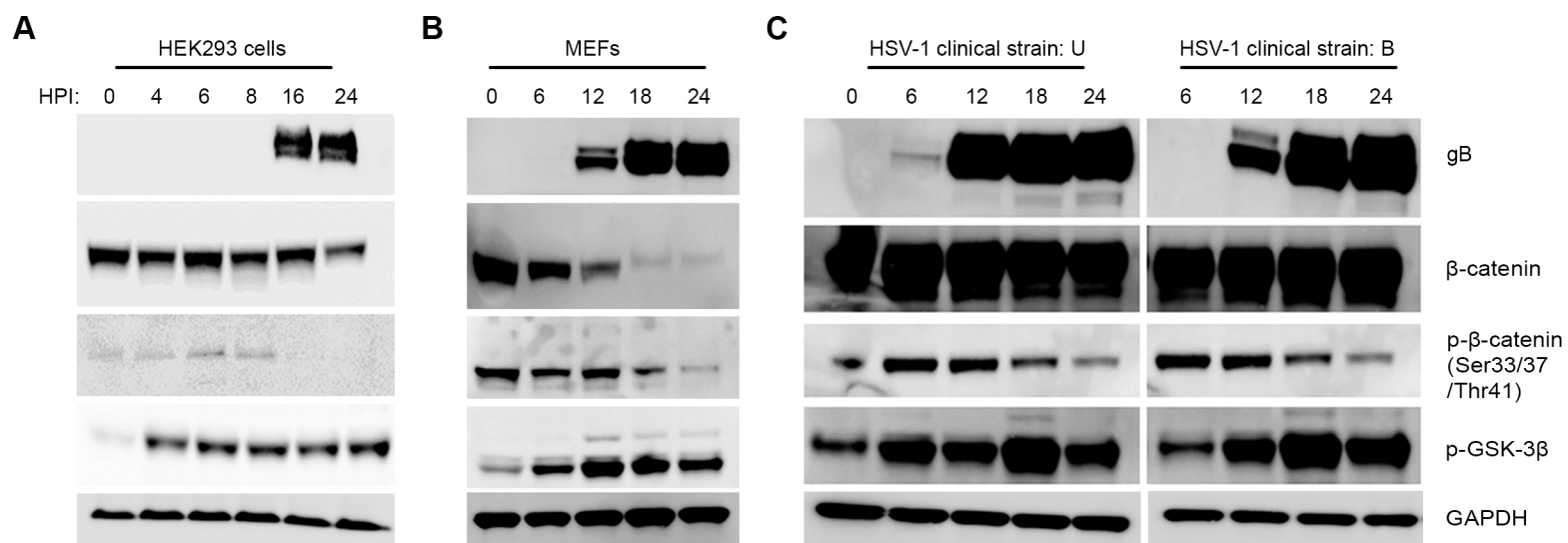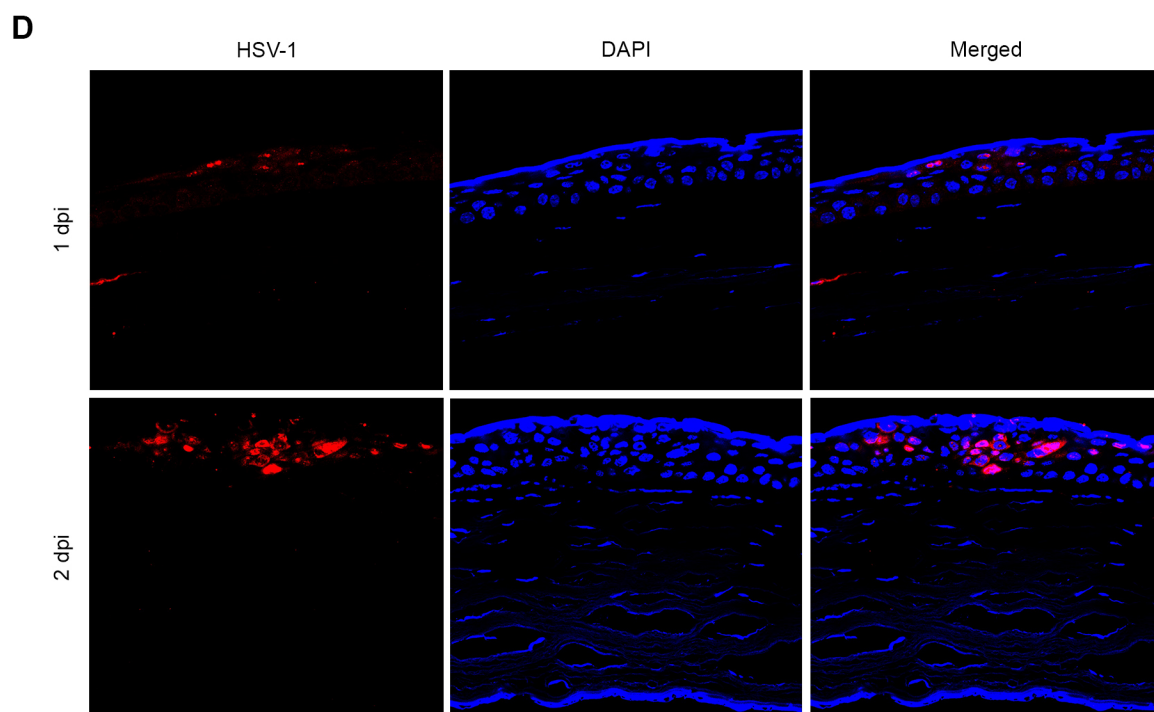

Supplement: FIG S1 [file mbio.02792-21-sf001.pdf]

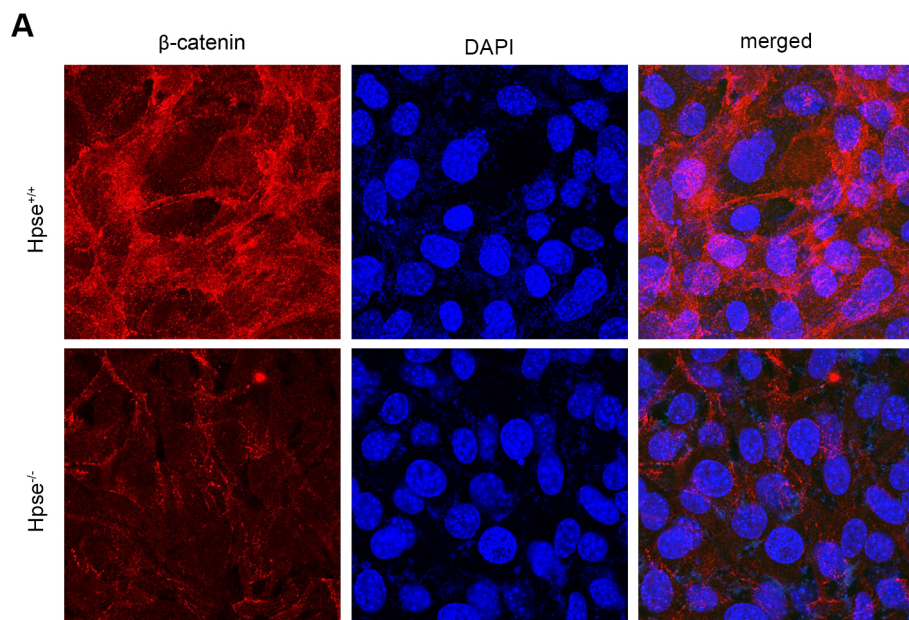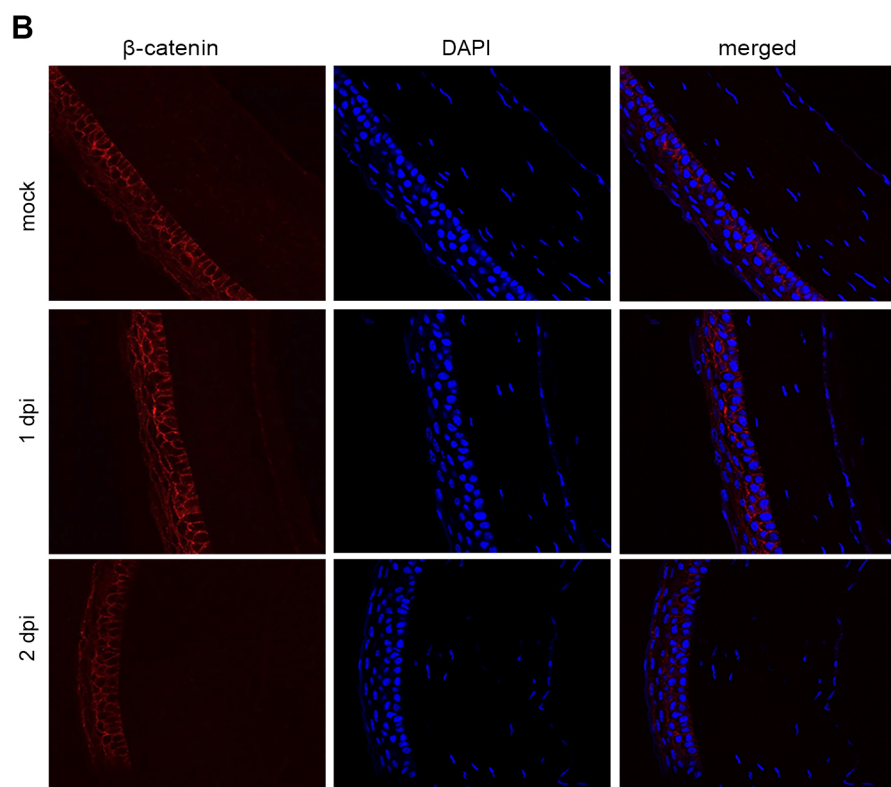

Hpse-KO ocular tissue

Supplement: FIG S2 [file mbio.02792-21-sf002.pdf]

**A**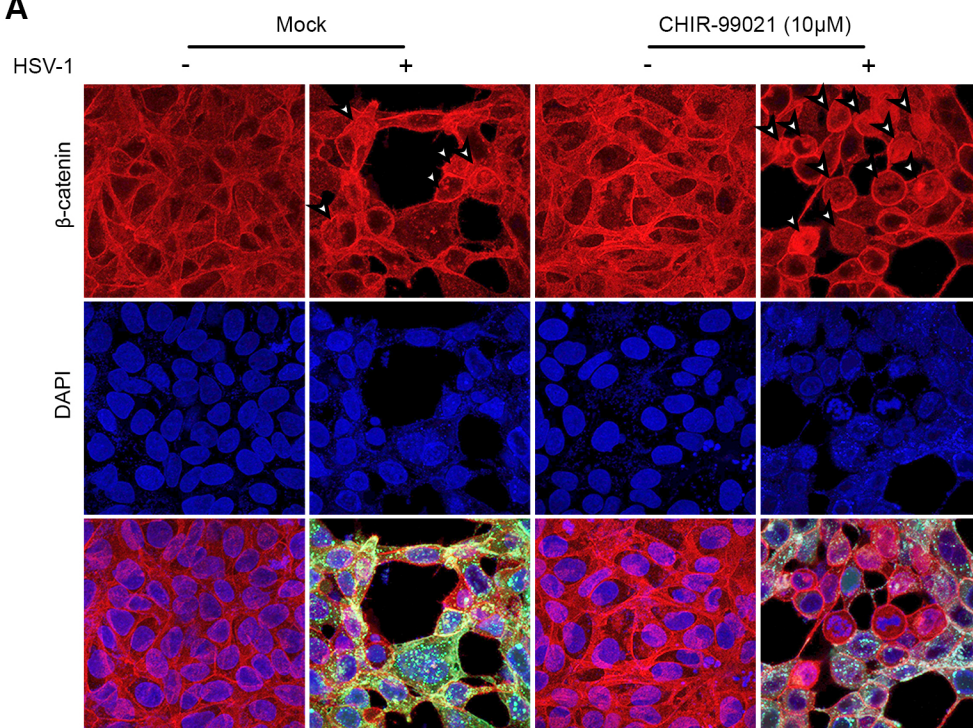

Supplement: FIG S3 [file mbio.02792-21-sf003.pdf]

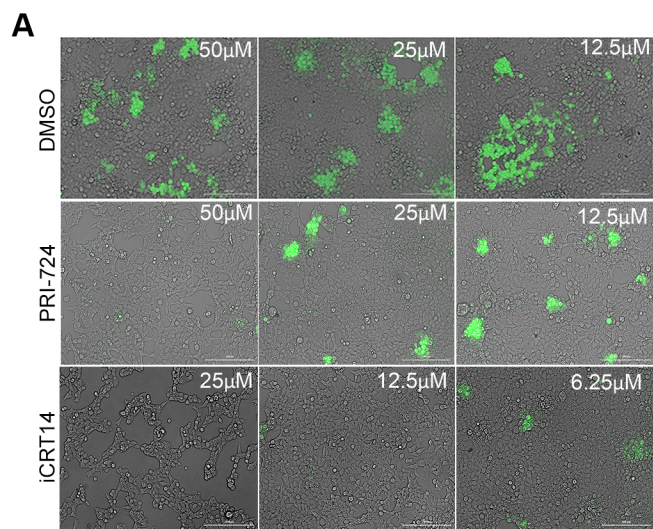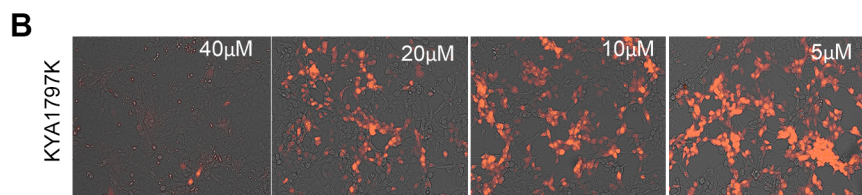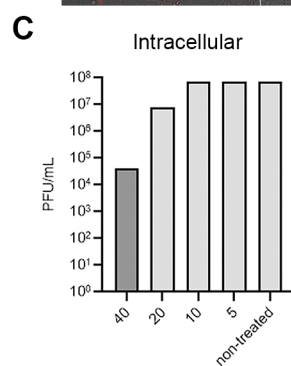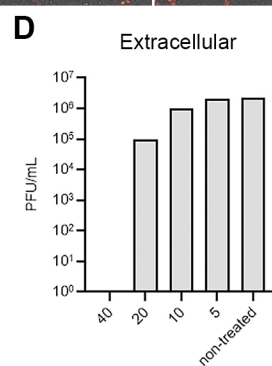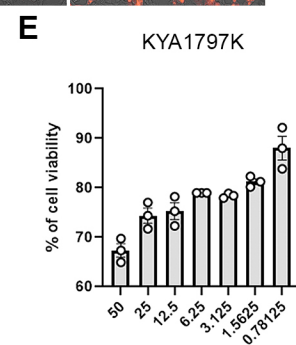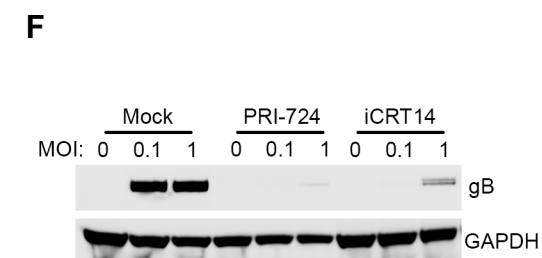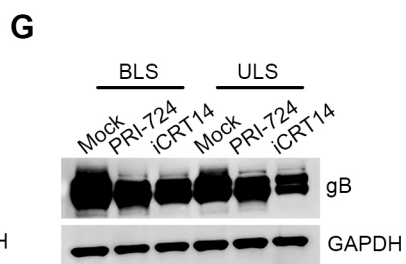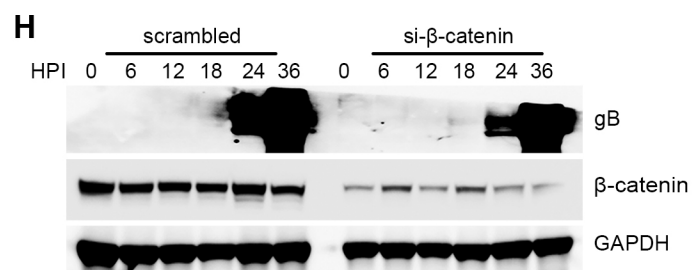

Supplement: FIG S4 [file mbio.02792-21-sf004.pdf]
